# Cooking, Food Agency and Pro- Health Behavior

**DOI:** 10.1007/s40572-026-00541-x

**Published:** 2026-05-16

**Authors:** Amy B. Trubek, Emily H. Belarmino, Julia A. Wolfson, Jacob Lahne

**Affiliations:** 1 Nutrition and Food Sciences, University of Vermont, 230 Marsh Life Sciences, 109 Carrigan Drive, 05405 Burlington, VT United States; 2 Departments of International Health and Health Policy and Management, Johns Hopkins Bloomberg School of Public Health, Baltimore, United States; 3 Food Sciences, Virginia Tech, Blacksburg, United States

**Keywords:** Food Agency, Food Environment, Cooking, Meals, Nutrition Security, Health

## Abstract

**Purpose of Review:**

We explore and summarize the evidence linking cooking to health with consideration of the broader implications for nutrition security. We begin with an overview of social transformations in everyday meal preparation over the past century and how these transformations and cooking at home are associated behaviors. With increased availability of prepared and processed foods in the food environment, making meals at home has emerged as a pro-health behavior with the potential to promote nutrition. Food agency is a means of defining and assessing meal preparation practices, which include cooking but *also* other practical and cognitive actions. We discuss the theory and measurement of food agency and provide a framework for action to better understand and address possible associations between food agency, cooking skills and behavior, and nutrition security.

**Recent Findings:**

Available evidence suggests higher level cooking skills, greater frequency of cooking at home, and higher cooking confidence are associated with greater fruit and vegetable intake and higher diet quality. However, numerous barriers to making meals from whole ingredients at home exist including food environments and sociocultural pressures that prioritize convenience.

**Summary:**

Much of the current research on food and cooking remains focused on *consumption.* However, more work is needed to articulate the pathways through which food agency and cooking could support dietary quality and nutrition security.

##  Introduction

This review examines food agency as a conceptual framework with the potential to help explain and address societal issues related to nutrition security, defined as consistent, reliable, and equitable access to nutritious foods that meet nutrient needs and support health and wellbeing. In brief, food agency characterizes the degree to which an individual is empowered to act effectively in setting and achieving food-provisioning goals (*including* cooking). As our understanding of planetary constraints grows, so too does the realization that current actions in food systems affect our future choices. Long term nutrition security can only be ensured if we build resilience into all areas of the food system, including diverse aspects of consumer behavior.

In the United States, substantial changes to food environments have been documented in the last century, especially the last fifty years, with consequences for diets, nutrition, and health. The modern food environment now provides numerous opportunities to purchase food already prepared and thus myriad options exists for others to do the work of planning, food selection, and meal preparation. For example, the United States Department of Agriculture Economic Research Service (USDA ERS) reports an increase in the proportion of total household food expenditures that come from restaurants and other away from home sources from 20% in 1940 to over 50% in 2023 [1]. Beyond purchasing meals away from home, the current food environment also provides a significantly expanded set of options for food already processed and prepared in some manner *before* purchase [2]. These foods can be purchased in numerous retail outlets, including grocery stores, convenience stores, restaurants, gas stations, etcetera. Large retail grocery stores are the greatest source of household calories (65%) for Americans. However, the fact that people shop in grocery stores for most of their calories is not an indicator that people are buying whole, scratch ingredients that will be then transformed into meals, due to other changes in the American food environment [3–5]. Ultra-processed foods (UPFs) now dominate US supermarket shelves and comprise more than 50% of Americans’ diets across all demographic groups, even when they cook frequently at home [6, 7]. UPFs are industrially produced foods containing little to no whole foods and are made mostly (or fully) from substances extracted from whole foods [8]. Additives such as colors, flavors, and emulsifiers are often added to improve the palatability of the final product. Examples of UPFs include savory snacks, soft drinks, cookies, candy, reconstituted meats like hot dogs, and pre-made meals like instant soups and TV dinners. Compared to unprocessed foods like fresh vegetables and fruits, UPFs cost considerably less per calorie ($0.55 vs. $1.45 per 100 kcal) [9]. While UPFs can be part of a nutritious diet, many are high in nutrients of concern such as added sugar, sodium, and saturated fat, and their high consumption could crowd out the intake of other nutrient-dense foods [7, 10]. In recent years, evidence linking UPF consumption and poor health outcomes has grown [11]. A consequence of these shifts in the food environment is that making meals at home is now a choice rather than an expectation and is most accessible to individuals with greater ability to make decisions and enact their preferences (i.e., greater agency).

The concept of food agency acknowledges a set of complex actions that impact what happens before food *consumption*, including often neglected yet crucial processes required for human nourishment: planning, shopping, and preparing food. In the context of intense focus on what people eat and how it impacts their health, defining and focusing on pre-consumption tasks (and the requisite skills needed to complete those tasks) uncovers unexamined assumptions, particularly an underestimation of the time and effort needed to be able to prepare a meal, even in the best of circumstances. Simultaneously, the ease by which one can ‘outsource’ many decisions and tasks through the purchase of pre-prepared and ultra-processed foods, consequentially, has impacted the exposure to and enactment of the daily intersection of cognitive, social, and physical abilities used to prepare meals at home [12]. Thus, perceptions of time poverty *and* structural constraints create impediments to meal preparation as do the widespread availability of food already prepared.

As Trubek et al. state: “The concept of food agency explains two contemporary realities for American home cooks: one, there is always an option not to cook and still be fed; and two, the efforts to successfully make a home cooked meal require more than mechanical skills but navigational ones too [13].” Thus, food environments, food agency and cooking shape an individual’s ability and aspiration *to produce* a meal thereby also shaping diets, nutrition, and health [13, 14]. To grasp the what, how and why of current food consumption trends, it is crucial to examine preparing meals, or the planning, provisioning and compiling of ingredients into dishes “as a skilled practice in relation to social and cultural contexts and constraints (13:298)”; thus, the array of activities required to make them must be acknowledged as part of current concerns about the health consequences of food consumption trends.

**Fig. 1:**
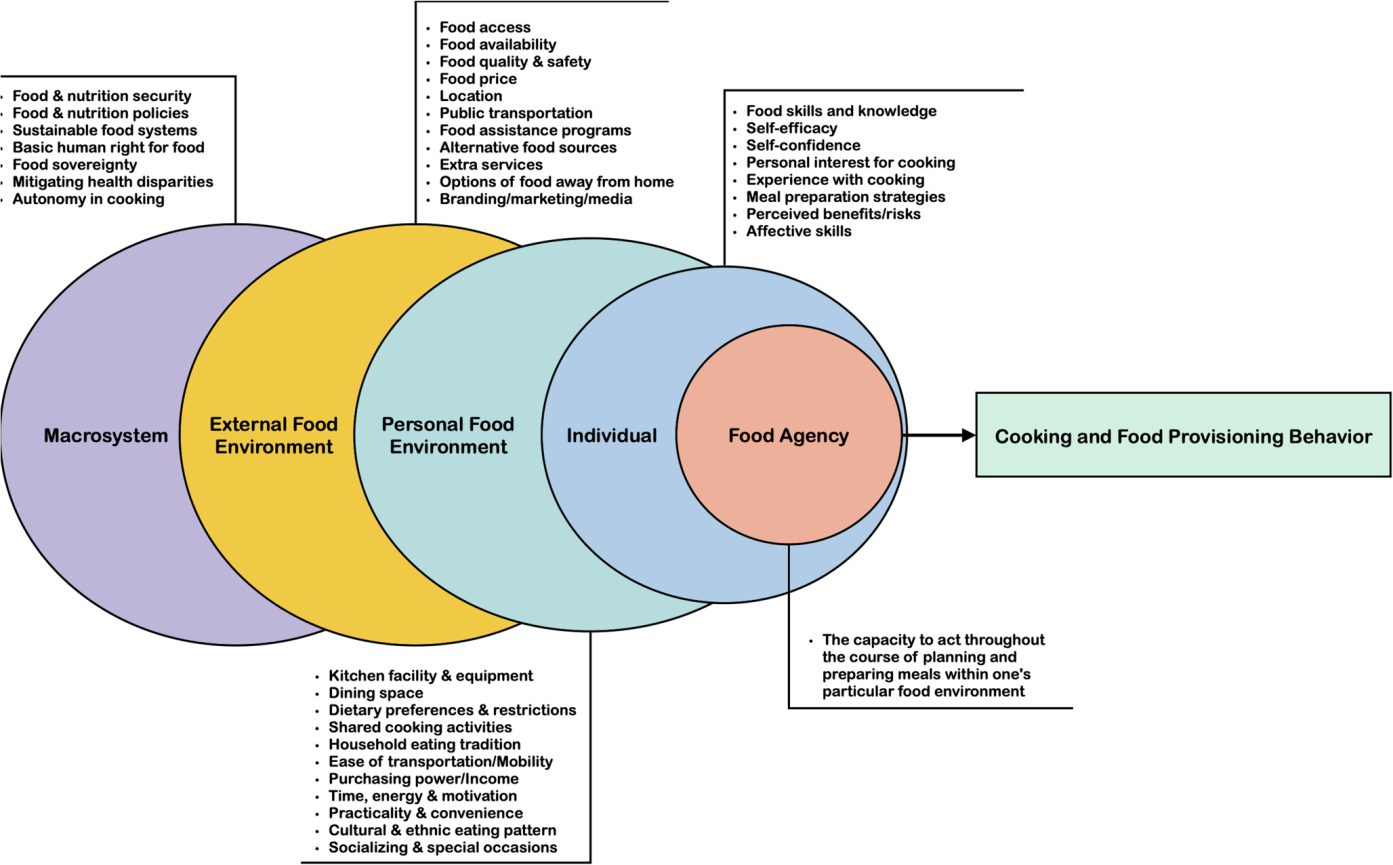
A conceptual model reflecting the relationship between food agency and cooking and food provisioning behavior [15]

In this narrative review, we consider food agency as a means to support and strengthen nutrition security in the United States and beyond, given the transformations in food environments along with changed consumption patterns. We highlight the concept of food agency - a theoretical and practical framework - and allied developed tools (a scale measure and a curriculum). Trubek et al. [13] and Wolfson et al. [14] propose “food agency” as a comprehensive framework for understanding cooking behavior and designing effective interventions that successfully transfer the range of cognitive, mechanical, sensorial, and navigational skills required for everyday meal preparation. Food agency is the capacity (encompassing a person’s skills, attitudes, and self-efficacy) to take those steps within one’s particular food environment and thus contribute to navigating it with success. Figure 1 (below) presents the interlocking and interdependent domains of knowledge, action and access involved for the final determination of any individual’s food consumption decisions.

## Shifts Away from Home Food Preparation

Numerous barriers to making meals from whole ingredients at home exist, including the access to and cost of such ingredients, perceived (or actual) time scarcity, knowledge and equipment gaps and lack of enjoyment regarding the cooking process [16, 17]. There are also sensory preferences for foods prepared by others and relief in not having to perform the complex tasks required. Everyone must eat each day, but in the current American food environment everyone does not need to make the food to be consumed; this has led to a “convenience orientation” that drives the organization of the food environment and everyday purchasing and consumption decisions [18].

Many American adults do not regularly cook at home. The long-term shift away from daily reliance on what can be called ‘traditional’ home cooking has links to women increasingly entering the workforce, especially since the 1970s. As more women joined the workforce outside the home, the prevailing social norms pressuring women to continue responsibility to prepare meals for their family have lingered, today now often connected to ideals related to healthy eating [19], but without the concomitant time or capacities as in previous eras. Women of color, single parents, and those of low socio-economic status often experience these pressures more acutely [19–22] and thus may be more inclined towards a “convenience orientation”. Age is another relevant factor. For example, one research study looked at women of varying ages (mean 32 years versus mean 68 years) and found that generational differences in home cooking practices were “related to more current lifestyle factors” (e.g., ordering take out) rather than divergent meal preparation skills [23]. Does this reveal a broader reality or not? Meanwhile, cooking skills in young adulthood have been found to predict better dietary behaviors and intake 10 years later [24].

Social determinants of health – or the social and environmental conditions that shape health outcomes – drive many food-related behaviors, including cooking behaviors. Socioeconomic status actively structures the choices around eating at home or relying on already prepared foods, but not unidirectionally. Access to grocery stores and other healthy food outlets, transportation options, affordability of nutritious options, particularly in comparison to less nutritious options, are all important factors that shape decisions about whether, what, and how individuals cook [25, 26]. As both Finley and Wolfson et al. point out, lower-income individuals who “always” cook may be making a “forced choice,” reflecting limited food budgets that also precluded buying costlier fresh and less processed ingredients [27, 28]. In a large survey on cooking practices [29], lower income individuals were more likely to state they cooked “always” or “never,” and higher income and higher educated individuals were more likely to state they cooked “sometimes.” These findings suggest that with higher incomes it is easier to cook less because those individuals have the disposable income to eat out more often. What remains to be better understood is the causal effect of these varying choices on overall dietary quality and measures of individual and population health. There are numerous complicating considerations when determinants of health are under evaluation; cooking “less” or “more” is not a singular claim nor insight into behavior. The answer depends on what people cook, and how they prepare it, and how much ultimately is consumed.

### Cooking as a Health Behavior

At the same time, consumption patterns are not static across a day, a week, or a month, and therefore efforts to change consumption patterns will need to consider that skills and knowledge are enacted at different levels of intensity across time (in a day or across a week). Transformations in the food environment and changes in home cooking behaviors have led to greater scrutiny – in research and through practice - on the associations between purchasing food away from home, availability and use of UPFs, time spent in active meal preparation and overall American dietary intake and dietary quality [30]. One area of interest is to wrest cooking from unexamined connections to “domestic life” or “women’s work” and to simply state, “cooking is a health behavior.” There are areas of inquiry among social scientists exploring the historical transformation of the food environment [20–22] as to the societal consequences - for example towards family cohesion and preservation of cultural heritage. However, within the robust area of research related to cooking as a health behavior, the goal is to ascertain whether (or not) cooking behavior is more optimal for *health*. Home cooked meals might have consequences for what people eat and consequently risk of diet-related cardiometabolic diseases such as cardiovascular disease, cancer, and diabetes.

In recent years, numerous culinary interventions with both children [31, 32] and adults [33, 34] have been developed with the intent of improving the quality of dietary intake, especially the increased consumption of whole fruits and vegetables. Such interventions appear to have a positive impact on cooking confidence and knowledge (in children and adults), and dietary intake, especially fruit and/or vegetable intake, among adults [34–37].

Nutrition, public health, and medical professionals increasingly recommend cooking at home to improve diet quality, keep caloric intake within recommended levels, and prevent (or treat) diet-related diseases [38–40]. Prominent examples include the recent rise of the culinary medicine movement in the last ten years aimed at integrating culinary arts, nutrition, and medicine to prevent and treat disease, and recommendations for cooking at home in the 2020–2025 Dietary Guidelines for Americans, the omnibus national policy directive on nutrition. As the evidence continues to grow, cooking programs appear to be a promising strategy to support nutrition security via improved knowledge, confidence, and skills with food.

Focusing on cooking as a health behavior means that the activities required in order to cook at home must be comprehensively understood and accurately measured to quantify its relationship to dietary intake and health outcomes, and to assess and develop interventions to effectively increase home cooking [41–43]. The impact is measured in relation to specific diet or nutrition outcomes [44]. However, there are multiple complex steps that powerfully impact the end point of consumption that are not as closely considered as crucial in the quest to change behavior [45]. An example of the self-efficacy that can emerge from higher food agency or food literacy (another framework used in public health and nutrition fields) is meal planning, a behavior associated with high rates of cooking food at home [13, 46]. The concept of “food literacy” [46] describes the multiple, knowledge-based skills necessary to “ensure regular food intake that is consistent with nutrition recommendations.” Food literacy does not address the capacity for the translation of *aspiration to action*, which does occur in the food agency framework [13, 14].

##  Food Agency

**Fig. 2:**
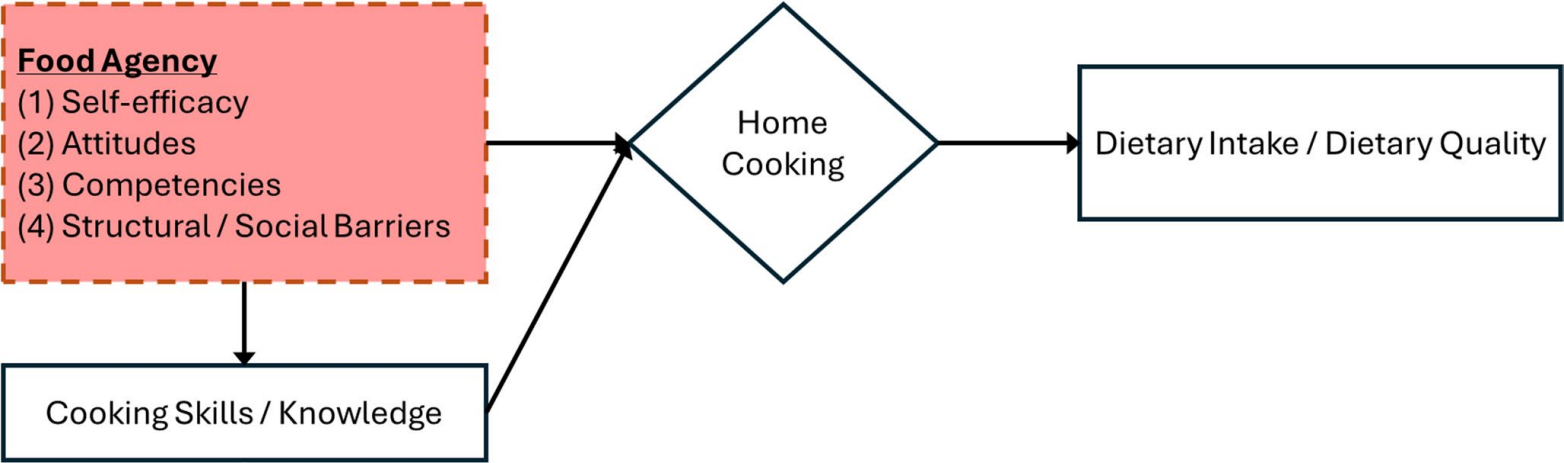
Framework connecting food agency and dietary intake and quality. Adapted from figure by Jiakun Yi (unpublished grant proposal)

While cooking-focused interventions show promise in improving dietary intake as well as cooking confidence and knowledge, the ability to cook is not the only skill needed to make nutritious meals and snacks. The more complex set of requirements is captured in the food agency framework (Fig. 2), which can help articulate both *what should happen (e.g. expert recommendations)* and *why it doesn’t always occur (poor uptake and compliance)*.

According to the food agency framework, food agency is based on a person’s [1] perception of their food preparation skills [2], attitude towards food and cooking [3], self-efficacy about achieving their food-related goals, and [4] experience with social obstacles and supports in their environment. The food agency approach contrasts with typical public health cooking and nutrition interventions which often focus on cooking demonstrations, specific recipes or technical skills. It embraces the integrated and interdependent factors of *context and capacity* – no singular cause and effect effort will transform behavior, at the individual or population level [47–49]. The concept of food agency also acknowledges the organization of the contemporary food system, which provides numerous pre-prepared food options so that individuals do not have to cook but can still be fed.

Having food agency means being *empowered* to cook and therefore being in possession of the full range of skills and capabilities from which daily meal preparation flows [13]. Food agency is grounded in robust theories as to the concurrent importance of individual self-efficacy and social and structural agency to change or expand decisions. Discrepancies and variance in levels of food agency can explain why some individuals set and achieve their cooking and food-related goals while others remain ambivalent and more likely to exploit the abundant alternatives to home meal preparation.

Food preparation frequency is positively correlated with cooking self-efficacy [14], a relationship that may be bi-directional. Conversely, lack of confidence can limit meal preparation at home. It may be the case that neither technical skills nor one’s ability to navigate their food environment may be sufficient – a positive attitude about cooking is crucial for people to prepare foods. From the view of agency theory in psychology, whether people think positively or negatively about their own abilities enhances or hinders those abilities. Studies have identified that skills and self-efficacy work in concert; the food environment and sociocultural and economic environment helps or hinders this relationship [50–53]. Food agency as a conceptual framework acknowledges the interdependent nature of cooking and food-provisioning behaviors, and thus while observable on the individual level, they are complex and culture-bound and emerge from the interplay of individual skills and attributes, circumstance, and social structural supports and barriers [52, 53]. In fact, the cognitive and practical capacities necessary for cooking reach beyond the kitchen to encompass a person’s ability to navigate their neighborhood food environment to shop for and procure food, budget appropriately, plan meals, manage food to avoid spoilage, manage time to fit meal preparation into their daily obligations, navigate social and cultural norms and expectations around food, and the technical skills to actually prepare food [20–22]. Of course, someone can have a high degree of food agency and continue to adopt a convenience orientation in consumption patterns. But without the capacity to enact these cooking and food provisioning behaviors it will be more difficult to achieve nutrition security.

## The CAFPAS scale: Measurement of Capacity

Therefore, food agency is a *holistic* framework that integrates the skills and knowledge Americans might employ in cooking and provisioning and it acknowledges the necessity for a *common*,* underlying capacity to effectively set and achieve cooking- and provisioning-related goals* (see Fig. 2). A companion survey tool, called the Cooking and Food Provisioning Action Scale (CAFPAS), was developed to measure an individual’s *capacity to effectively set and achieve cooking- and provisioning-related goals* [16]. This valid and reliable measurement tool [16] operationalizes the different dimensions of engagement with food required for home cooking, thus it is possible to assess Americans’ cooking behaviors and relate them to health outcomes and assess the impact of interventions to increase home-cooking. Until recently, survey instruments have asked about general cooking confidence, or specific cooking techniques (such as poaching fish or making specific sauces) or general food knowledge to evaluate cooking behaviors and skills. However, there is no good evidence that specific types of activities are axiomatic as to what defines “cooking” and thus necessary or sufficient to fulfilling an abstract definition used in survey research, which is why reported or observed self-efficacy in the context of certain cooking activities might be a more robust measure, especially when linked to health outcomes.

As Lahne et al. point out in relation to the design of this scale as compared to others used to examine allied food and nutrition issues: “Agency is a framework, therefore, that is consistent with recent research that examines food-related knowledge and skill as more than rote, mechanical action like “Food Literacy” [16]. Food agency is an appropriate, consensus approach for researchers and practitioners seeking to promote cooking as a health behavior. Since 2017, CAFPAS has been successfully used for measurement in diverse studies (across multiple countries), that seek to relate food agency to various dietary-quality outcomes and has emerged as an extended scholarly and practice network across the United States and beyond [54–57]. For example, the CAFPAS has been used (in English) for cooking and nutrition research in the United States, Britain, Denmark, Ireland, Australia, and Canada, and has been translated into multiple languages (including French, Swedish, Spanish, Korean, Hebrew and Czech) for use with non-English speaking populations. Higher food agency, as measured by the CAFPAS, has been associated with more frequent instances of home cooking and scratch cooking, and less frequent cooking with packaged ingredients [58]. Additionally, higher food agency has also been positively associated with food security and vegetable consumption [59–61]. Further research is needed from longitudinal studies and intervention trials to determine whether any or all of these associations are causal in nature or persistent over time [62–66].

## Conclusion

Everyday meal preparation is increasingly identified as optimal by nutrition and health professionals, including in order to fulfill the current national guidelines related to healthy eating (). When it comes to the challenges related to improving health behaviors and nutrition security in the United States, there are clear impediments – social, structural, economic, environmental – to realizing such aspirations [64–67]. In much of the health and wellness space, the standard measures to ascertain achievements tend to revolve around individual measures: BMI, blood glucose levels, diet quality scores. The practical realities of what people must do to plan, source, prepare and only then put food on the table (or decide not to participate in some or all those actions) and how that shapes their dietary intake and diet quality are given considerably less attention. And there are numerous directions for study design, data collection, data analysis and the connection of research results to proposed interventions, at the level of individual or population health [66, 68, 69, 72], for example interventions based in teaching the appropriate confidence and skills that involve long-term data collection as to persistence of practice and impacts on nutrition security and health outcomes. The food-agency framework supports the commonsense, empirical observation that *there are many effective*,* different routes by which an individual can provision or cook for themselves in a healthy fashion*, shaped by culture, society, economy and by the specific contexts in which they live. And yet it continues to be difficult to enact such routes [70, 71, 73, 74]. The concept of food agency integrates the food environment, the social determinants of meal preparation and individual capacities. Food agency acknowledges the need to move beyond assumptions about individual consumption patterns and overall food consumption trends in the United States. The food agency conceptual framework seeks to address, holistically, the interrelated factors that shape food procurement and preparation decisions. Such an understanding is necessary to develop policies and programs to improve the food system and food environment such that it promotes food and nutrition security, and healthy and sustainable diets for all.

## Key References

- Trubek AB, Carabello M, Morgan C, Lahne J. Empowered to cook: The crucial role of ‘food agency’ in making meals. Appetite [Internet]. 2017;116:297–305. Available from: 10.1016/j.appet.2017.05.017.
  - ○ This article provides the theoretical foundation for the concept of food agency.
- Wolfson JA, Bostic S, Lahne J, Morgan C, Henley SC, Harvey J, et al. A comprehensive approach to understanding cooking behavior. BFJ [Internet]. 2017;119(5):1147–58. Available from: 10.1108/bfj-09-2016-0438.
  - ○ This article provides the rationale for researching cooking behavior in relation to nutrition and public health.
- Lahne J, Wolfson JA, Trubek A. Development of the Cooking and Food Provisioning Action Scale (CAFPAS): A new measurement tool for individual cooking practice. Food Quality and Preference. 2017;62:96–105. Available from: 10.1016/j.foodqual.2017.06.022.
  - ○ This article provides processes and results involved in the creation of the validated measurement of food agency using the Cooking and Food Provisioning Action Scale (CAFPAS).
- Bowen S, Brenton, J, Elliott S. Pressure Cooker. Oxford: Oxford University Press; 2019.
  - ○ This peer-reviewed scholarly monograph integrates qualitative research and theoretical analyses to synthesize current issues related to socioeconomic status, gender, and capacities to produce meals deemed healthy.
- Wolfson JA, Lahne J, Raj M, Insolera N, Lavelle F, Dean M. Food agency in the United States: associations with cooking behavior and dietary intake. Nutrients [internet]. 2020;12:877. Available from: 10.3390/nu12030877.
  - ○ This article examines the relationship between food agency and several food- and cooking-related behaviors among two samples: a national sample of US adults and a sample of parents of 2-9 year old children.

## Data Availability

No datasets were generated or analysed during the current study.
